# Age-Dependent Increase in Schmidt-Lanterman Incisures and a Cadm4-Associated Membrane Skeletal Complex in Fatty Acid 2-hydroxylase Deficient Mice: a Mouse Model of Spastic Paraplegia SPG35

**DOI:** 10.1007/s12035-022-02832-4

**Published:** 2022-04-20

**Authors:** Silvia Jordans, Robert Hardt, Ivonne Becker, Dominic Winter, Lihua Wang-Eckhardt, Matthias Eckhardt

**Affiliations:** 1 Present Address: Department for Pediatric Hematology and Oncology, Center for Pediatrics, University Hospital Bonn, Venusberg-Campus 1, 53117 Bonn, Germany; 2 Institute of Biochemistry and Molecular Biology, Medical Faculty, University of Bonn, Nussallee 11, 53115 Bonn, Germany

**Keywords:** Myelin, Schmidt-Lanterman incisure, Spastic paraplegia, Sphingolipid

## Abstract

PNS and CNS myelin contain large amounts of galactocerebroside and sulfatide with 2-hydroxylated fatty acids. The underlying hydroxylation reaction is catalyzed by fatty acid 2-hydroxylase (FA2H). Deficiency in this enzyme causes a complicated hereditary spastic paraplegia, SPG35, which is associated with leukodystrophy. Mass spectrometry-based proteomics of purified myelin isolated from sciatic nerves of *Fa2h*-deficient (*Fa2h*^−/−^) mice revealed an increase in the concentration of the three proteins Cadm4, Mpp6 (Pals2), and protein band 4.1G (Epb41l2) in 17-month-old, but not in young (4 to 6-month-old), *Fa2h*^−/−^ mice. These proteins are known to form a complex, together with the protein Lin7, in Schmidt-Lanterman incisures (SLIs). Accordingly, the number of SLIs was significantly increased in 17-month-old but not 4-month-old *Fa2h*^−/−^ mice compared to age-matched wild-type mice. On the other hand, the relative increase in the SLI frequency was less pronounced than expected from Cadm4, Lin7, Mpp6 (Pals2), and band 4.1G (Epb41l2) protein levels. This suggests that the latter not only reflect the higher SLI frequency but that the concentration of the Cadm4 containing complex itself is increased in the SLIs or compact myelin of *Fa2h*^−/−^ mice and may potentially play a role in the pathogenesis of the disease. The proteome data are available via ProteomeXchange with identifier PXD030244.

**Supplementary Information:**

The online version contains supplementary material available at 10.1007/s12035-022-02832-4.

## Introduction

Schmidt-Lanterman incisures (SLIs), also known as myelin incisures or Schmidt-Lanterman clefts, are cytoplasmic channels of the Schwann cells in the myelin internodes. It is generally assumed that SLIs facilitate transport of metabolites, ions, and signaling molecules between peri-nuclear and adaxonal cytoplasmic regions by reducing diffusion distances because of radial diffusion through gap junctions [1].

Galactosylceramide and its sulfated derivative sulfatide are abundant sphingolipids in the nervous system [2]. A large percentage of galactosylceramide and sulfatide in CNS and PNS myelin of mammals contains 2-hydroxylated fatty acyl residues [3, 4]. In myelinating cells, the 2-hydroxylation reaction is exclusively catalyzed by the enzyme fatty acid 2-hydroxylase (FA2H), a cytochrome b5 domain-containing enzyme of the endoplasmic reticulum [5, 6]. Although free fatty acids are substrates for the enzyme in an in vitro activity assay [7], X-ray structural analyses suggest that ceramides may be additional in vivo substrates [8]. The functional role of the 2-hydroxylation modification of sphingolipids is not fully understood. Hydroxylated sphingolipids appear to have unique roles in signal transduction [9] and may affect the turnover of membrane proteins by their influence on the mobility of lipids in membrane subdomains (or lipid rafts) [10–12].

Mutations in the *FA2H* gene that reduce or abolish activity of the enzyme cause a complicated form of hereditary spastic paraplegia type 35 (SPG35) associated with leukodystrophy, which is also known as fatty acid hydroxylase-associated neurodegeneration (FAHN) and as a subtype of neurodegeneration with brain iron accumulation (NBIA) [13]. More than 40 disease-associated human *FA2H* mutations have been reported [14]. *Fa2h*-deficient (*Fa2h*^−/−^) mice serve as animal model of SPG35/FAHN and develop a phenotype that is reminiscent of symptoms of the human disease [15, 16]. In a previous study, we found evidence for alterations in the CNS myelin proteome of *Fa2h*^−/−^ mice [17]. Although SPG35, like hereditary spastic paraplegias in general, is characterized by degeneration of upper motor neurons, peripheral neuropathy has been described in about 30% of the patients [14]. Karle et al. [18] estimated a prevalence of peripheral neuropathy of about 60% in all cases of hereditary spastic paraplegia together. In the present report, we performed a myelin proteome study of sciatic nerves, in order to examine possible molecular changes in the PNS myelin of *Fa2h*^−/−^ mice.

## Experimental Procedures

### Animals

Generation of *Fa2h*^−/−^ mice (*Fa2h*^tm1Meck^; MGI:3829000) and genotyping has been described previously (Zöller et al., 2008). Animal experiments have been approved by the national authorities (reference number: 84–02.04.2014-A039).

### Antibodies

Antibodies used in this study are listed in Table 1 and were kind gifts from Peter Prophy and Arthur M. Butt or were purchased from the following companies: Abcam (Cambridge, UK), Antibodies Incorporated (Davis, California, USA), Biorbyt (Cambridge, UK), GeneTex (Irvine, California, USA), Jackson ImmunoResearch (Philadelphia, Pennsylvania, USA), Merck (Darmstadt, Germany), and Thermo Fisher (Waltham, Massachusetts, USA).

**Table 1:** Antibodies used in this study

Antibody (host)	RRID	Order #	Lot #	Company/Source	Dilution
Caspr (rabbit)	RRID:AB_869934	ab34151	GR259109-3	abcam	1:1000 (IF)
CNP (mouse)	RRID:AB_2082608	MAB326	2,043,906	Merck	1:3000 (WB)
EPB41L2 (band 4.1G) (rabbit)	n/a	orb213897	DF2510	Biorbyt	1:500 (WB)
Lin7a (rabbit)	RRID:AB_10731933	GTX117114	40,471	GeneTex	1:3000 (WB); 1:100 (IF)
L-MAG (rabbit)	n/a	n/a	n/a	gift from A.M. Butt	1:5000 (WB)
MBP (rabbit)	RRID:AB_11211843	AB980	2,739,500	Merck	1:10,000 (WB)
Periaxin N-terminus (rabbit)	n/a	n/a	n/a	gift from P. Brophy	1:20,000 (WB)
SynCAM4 (Cadm4) (mouse)	RRID:AB_10673109	73–247	4437-4VA-37	Antibodies Incorporated	1:250 (WB); 1:100 (IF)
VAM1 (Mpp6; Pals2) (rabbit)	RRID:AB_1241437	GTX108010	39,721	GeneTex	1:1000 (WB)
anti mouse peroxidase (goat)	RRID:AB_2338503	115–035-044	132,855	Jackson ImmunoResearch	1:20,000 (WB)
anti-rabbit peroxidase (goat)	RRID:AB_2313567	111–035-003	137,093	Jackson ImmunoResearch	1:20,000 (WB)
anti-rabbit Cy3 (goat)	RRID:AB_2338006	111–165-144	143,018	Jackson ImmunoResearch	1:300 (IF)
anti-mouse Alexa Fluor 647 (goat)	RRID:AB_2535804	A21235	1,764,240	Thermo Fisher	1:300 (IF)

Abbreviations: *WB* Western blot, *IF* immunofluorescence

### Purification of Myelin from Sciatic Nerves

Myelin from sciatic nerves was isolated according to Caroni and Schwab [19] with the following modifications. Sciatic nerves were removed from mice that had been killed by cervical dislocation and stored at − 80 °C, before they were homogenized in isotonic 9.2% sucrose solution using a Dounce homogenizer (pooled nerves from one mouse). Myelin was then purified by sucrose density step gradient (9.2% and 28.4% sucrose) centrifugation. Myelin isolated from the interphase was washed with water, resuspended in 1-mM EDTA, and stored at − 80 °C.

### Lipid Extraction and Thin Layer Chromatography

Total lipid extracts from sciatic nerves were prepared as described [20]. Briefly, nerves were homogenized in methanol using a Dounce homogenizer, and then chloroform and 1% HClO_4_ were added to obtain a final ratio of 1:1:0.9 (chloroform/methanol/HClO_4_; v/v/v). Samples were mixed and centrifuged to facilitate phase separation. The organic phase was dried in a vacuum centrifuge, dissolved in chloroform/methanol (1:1; v/v), and sonicated (5 min) in a sonication water bath. Aliquots of the lipids were administered to silica gel 60 HPTLC plates (Merck) and separated in a solvent-saturated chromatography tank using chloroform/methanol/water (70:30:4; v/v/v) as solvent system. Lipids were stained by spraying TLC plates with a solution of 625-mM cupric sulfate, 8% phosphoric acid, followed by heating to 150 °C for 5 min [21].

### SDS-PAGE and Silver Staining

SDS-PAGE (10% or 12.5% acrylamide) under reducing conditions was performed as described [17]. Silver staining was performed according to Heukeshoven and Dernick [22].

### Western Blot Analysis

Semi-dry Western blotting on 0.1-µm nitrocellulose membranes (GE Healthcare, Chicago, Illinois, USA) was performed as described [17] using the transfer buffer 25-mM Tris, 192-mm glycine, 20% methanol, and pH 8.3 [23]. Transfer conditions were 25 V, 30 min or 2 mA/cm^2^, 70 min. Transfer efficiency was monitored by Ponceau S staining [24]. Membranes were blocked in 3% milk, 0.05% Tween 20, 20-mM Tris–HCl, 150-mM NaCl, and pH 7.4 and stained with primary antibodies in blocking solution (4 °C, overnight), followed by appropriate peroxidase-conjugated secondary antibodies (see Table 1 for antibodies used and dilutions).

### Mass Spectrometry and Data Analysis

Tandem mass tag 6-plex (TMTsixplex) labeling and liquid chromatography-tandem mass spectrometry (LC–MS/MS) measurements were performed as described previously [17]. Briefly, purified myelin samples were delipidated by acetone precipitation and then subjected to RapiGest (Waters, Milford, Massachusetts, USA) assisted tryptic digestion (enzyme to protein ratio = 1:100) including cysteine reduction and alkylation. Afterwards, peptides were labeled using TMTsixplex Isobaric Label Reagent (Thermo Fisher) and then combined into three labeling pools, with each pool containing one independent biological replicate of 6-, 13-, and 17-month-old wild-type and *Fa2h*^−/−^ mice. Thereafter, RapiGest was precipitated, samples desalted by solid-phase extraction, and each sample pool subjected to 12 well OFFGEL fractionation. Finally, all peptide fractions were separated by reversed phase chromatography (self-packed column: 100 µm × 200 mm, Magic C18 AQ, 5 µm, Bruker, Bremen, Germany) using an Easy-nLC 1000 UHPLC (Thermo Fisher) and analyzed by a data-dependent TOP10 method using a LTQ Orbitrap Velos mass spectrometer (Thermo Fisher). Raw files were processed with Proteome Discoverer 2.5 (Thermo Fisher) in combination with a Mascot 2.6.1 (Matrix Science, London, UK) search engine. Initially, MS1 precursor masses were recalibrated with the Spectrum Files RC node (Tolerances MS1/2: 20 ppm/0.02 Da) using a non-linear regression model. Spectra were searched against a Swissprot Mus musculus proteome database (downloaded 03/2021, 17085 entries) and two common contaminants database, cRAP (https://www.thegpm.org/crap/) and MaxQuant-contaminants (https://maxquant.org), in a reverse decoy approach. The enzyme specificity was Trypsin/P with up to two missed cleavages allowed. TMTsixplex was set as quantification method. Modifications were propionamide (C) and TMT (K, Peptide N-term) as fixed and oxidation (M) and acetylation (Protein N-term) as variable. Search tolerances were 20 ppm both for MS1 and MS2. Identified spectra (PSM) were validated by the Percolator node based on q-values to target false discovery rates (FDRs) of 1%/5% FDR (strict/relaxed). All spectra not passing the stricter FDR were submitted to a second-pass Mascot search employing relaxed parameters:

- Enzyme name: SemiTrypsin
- Max. missed cleavages: 1
- Dynamic mods: oxidation (M), acetylation (Protein N-term), propionamide (C), TMT (K, Peptide N-term)

After PSM validation by Percolator, the combined PSMs were aggregated to peptides and proteins according to the principle of strict parsimony and finally filtered at 1% peptide and protein FDR. In addition, the protein list was filtered to contain only master proteins. For quantification, TMT reporter ion signals were extracted at the MS2 level with 20 ppm tolerance using the most confident centroid. From this, the relative peptide/protein quantification was achieved using the following parameters:

- Peptides to use: unique + razor
- Reporter abundance based on intensity
- Co-isolation threshold: 30%
- Average reporter S/N: 10.

The resulting protein list was filtered for master proteins only and exported to R Studio (R version 4.1.0) for data processing, differential expression analysis, and visualization using the following additional packages: BioVenn 1.1.3, dplyr 1.0.8, EnhancedVolcano 1.12.0, ggplot2 3.3.5, ggrepel 0.9.1, limma 3.50.0, and pheatmap 1.0.12. First, contaminating proteins were removed including proteins labeled “Ig.” After log(2) transformation, data was filtered for proteins containing three intensity values for each age and genotype. The filtered data was then normalized by cyclic loess normalization, and differentially abundant proteins were determined by a moderated *t* test based on linear models (limma trend) [25]. To account for batch effects of individual TMT batches, batch was included as co-variate in the linear model. Finally, contrast for all relevant comparisons were extracted from the linear model and exported to individual result tables. Note that proteins with an absolute log(2)-fold change > 0.585 and a false discovery rate (FDR) < 0.1 were deemed significantly regulated. The mass spectrometry proteomics data have been deposited to the ProteomeXchange Consortium via the PRIDE [26] partner repository with the dataset identifier PXD030244 and 10.6019/PXD030244.

### Teased Fiber Preparation and Immunostaining

Sciatic nerves were immersion fixed in 4% paraformaldehyde in PBS, washed with PBS, and stored at 4 °C in PBS containing 0.02% sodium azide. Teased fibers were prepared as described [27] and dried overnight at room temperature. For quantification of SLIs, teased fibers were stained with Atto488-labeled phalloidin (Sigma-Aldrich, St. Louis, Missouri, USA). For immunofluorescence staining, teased fibers were post fixed and permeabilized in − 20 °C cold methanol for 15 min. After blocking with 2% bovine serum albumin and 0.3% Triton X-100 in PBS at 25 °C for 2 h, specimens were incubated with primary antibodies overnight at 4 °C in a moisturized chamber. After washing 6 times with PBS, specimens were stained with the appropriate secondary antibodies for 2 h at 25 °C (see Table 1 for antibodies and dilutions). Microscopic pictures were captured using an Axiovert 200 M microscope fitted with a Colibri LED system (Carl Zeiss, Jena, Germany). Length of nodes of Ranvier and paranodes in microscopic pictures were measured using the ZEN 3.2 software or Axiovision SE64 Rel. 4.9.1 (both from Carl Zeiss).

### Statistical Analysis

Data are presented as mean ± standard deviation (SD). Data were tested for normal distribution by Shapiro–Wilk test. Normal distributed data were analyzed by Student *t* test. A *p* value < 0.05 was considered statistically significant. In case of multiple comparisons, the FDR was controlled according to Benjamini and Hochberg [28]. For the proteome analysis, a FDR of 0.1 was chosen because of the exploratory nature of the study; otherwise the FDR was controlled at level 0.05.

## Results

### Proteome Analysis of Sciatic Nerve Myelin from Fa2h^−/−^ Mice Showed Increased Levels of Four Proteins that Are Known to Form a Complex in SLIs

**Fig. 1:**
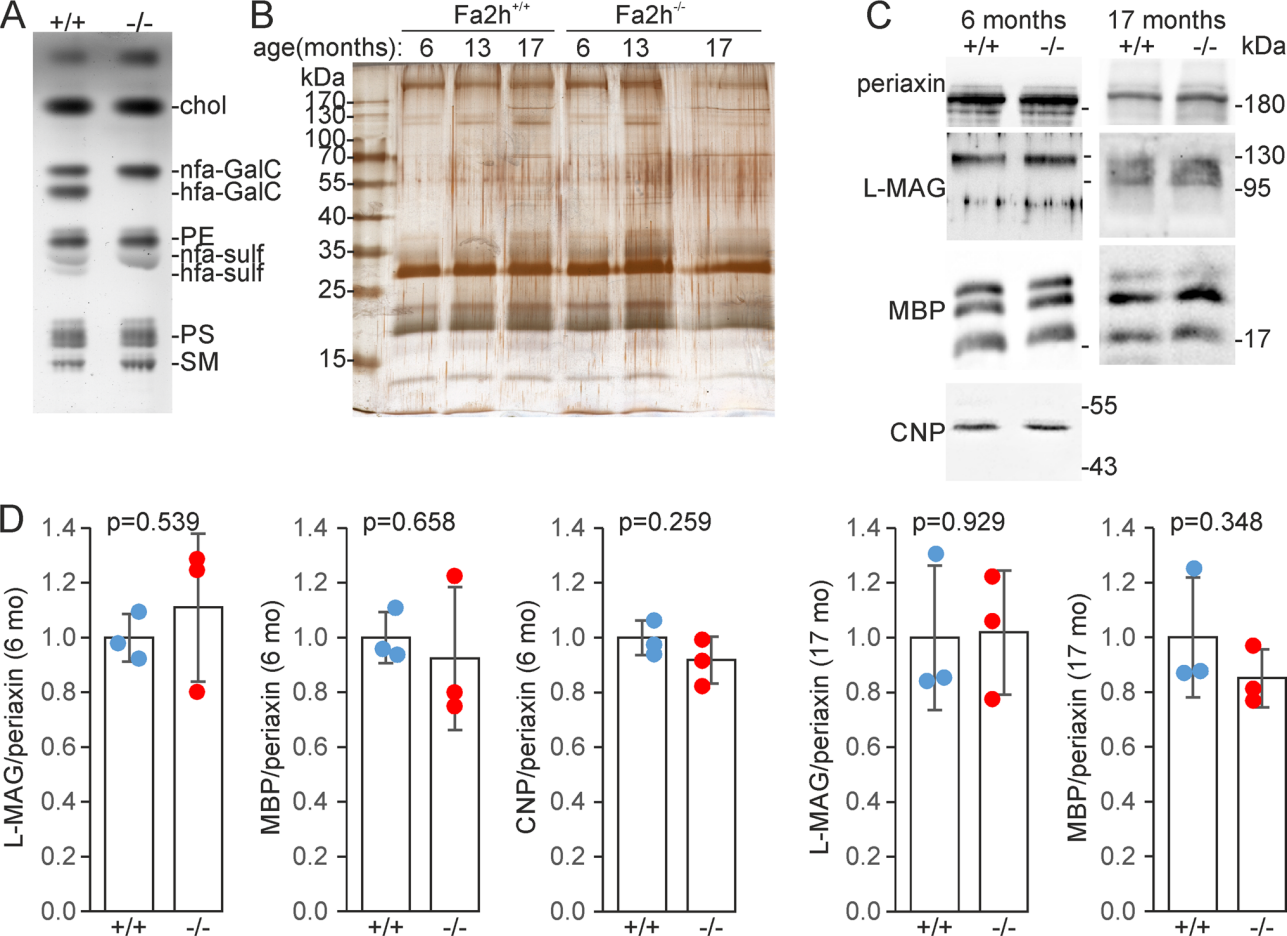
Sciatic nerve myelin purification. **A** Lipid thin layer chromatography of total lipid extracts isolated from purified myelin confirmed absence of 2-hydroxylated fatty acid containing galactosylceramide (hfa-GalC) and sulfatide (hfa-sulf) in *Fa2h*^−/−^ mice (nfa, non-hydroxylated fatty acid). **B** Representative silver staining of purified PNS myelin samples separated by SDS-PAGE. **C** Western blot analysis of purified myelin samples from 6 and 17-month-old mice showed comparable levels of major PNS myelin proteins: CNP, L-MAG, Periaxin, and MBP. **D** Densitometric quantification of Western blots. Intensities were normalized to periaxin. Data shown are the mean ± SD (*n* = 3 mice per genotype) with the mean of wild type set to 1; *p* values for *t* test

Myelin was isolated from sciatic nerves of *Fa2h*^−/−^ and *Fa2h*^+/+^ mice (aged 6, 13 and 17 months) by sucrose density gradient centrifugation. Purification and comparability of myelin samples were monitored by lipid analysis (Fig. 1a) and SDS-PAGE followed by silver staining (Fig. 1b). In line with previous analyses of sciatic nerves [15], 2-hydroxylated galactosylceramide and sulfatide were absent from *Fa2h*^−/−^ mice, whereas their non-hydroxylated isoforms were increased, resulting in only small changes in the levels of total galactosylceramide and sulfatide (Fig. 1a). In addition, levels of major myelin proteins were examined by Western blotting (Fig. 1c), which showed comparable concentrations of periaxin, 2',3'-cyclic nucleotide 3'-phosphodiesterase (CNP), large isoform of myelin-associated glycoprotein (L-MAG), and myelin basic protein (MBP).

**Fig. 2:**
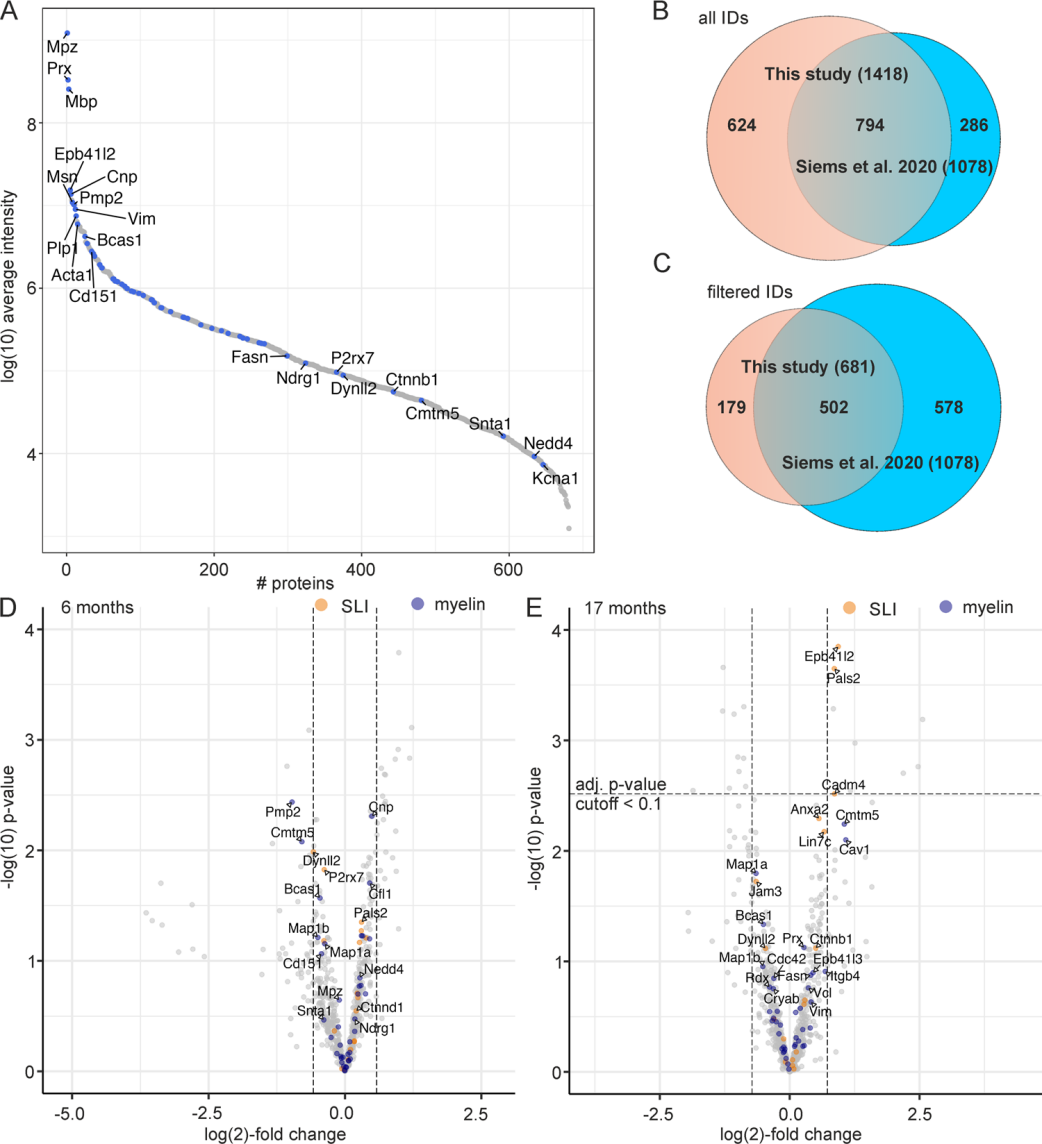
Myelin proteome analysis of sciatic nerves isolated from *Fa2h*^−/−^ mice. **A** Protein abundance plot showing the log(10) intensity for all 681 proteins that could be identified in all samples averaged over all samples. Known myelin proteins are highlighted in blue and a selection is labeled with their respective gene name. **B** Venn diagram comparing the PNS myelin proteins identified (in one replicate) in this study with the data published in Siems et al. [28] (dataset UDMS^E^, Fif1-data1-v1, 1). **C** Venn diagram comparing the PNS myelin proteins reliably identified (filtered IDs, abundance values in all replicates) in this study with the data published by Siems et al. [28] (dataset UDMS^E^, Fif1-data1-v1, 1). **D**, **E** Vulcano plot of myelin proteome data from 6-month-old (D) and 17-month-old mice (E). Data points show the -log_10_ transformed p-value versus median of the log_2_(fold change) (+ = increased in *Fa2h*^−/−^;—= decreased in *Fa2h*^−/−^) for proteins identified in all replicates. Proteins known to be present in SLIs are highlighted in orange and other known myelin proteins (according to Patzig et al. [29]) in blue. Selected myelin proteins are labeled with the corresponding gene names. Vertical broken lines indicate the 0.666- or 1.5-fold change thresholds. The horizontal line in (E) indicates the significance threshold when the FDR was controlled at level 0.1

Mass spectrometry of three biological replicates per age group was performed as previously described [17]. In total, 1418 protein groups could be identified in at least one biological replicate, and 937 protein groups could be identified in at least two replicates per age group (data have been deposited at the PRIDE partner repository with the dataset identifier PXD030244 and 10.6019/PXD030244). Major myelin proteins showed high abundance, as expected (Fig. 2a). After filtering the data to remove all protein groups not identified in all data sets, 681 proteins remained for quantitative evaluation (Supplementary Table S1). Compared with a list of 90 well-known myelin proteins identified in previous PNS myelin proteome studies [29, 30], our approach identified 59% of them in all samples. When compared with the myelin proteome data set published by Siems et al. [28], 74% of those proteins could be identified in our study and 47% were identified and quantified in all samples (Fig. 2b, c). Samples from 13-month-old mice were excluded from further analysis, because cluster analysis and principal component analysis revealed inconsistencies in the results for this age group with these samples for unclear reasons (supplementary Fig. S1).

We then tested for significantly altered proteins using a moderated t-test based on linear models (limma). After correction for multiple comparisons, significant changes (using the criteria: |log(2)(fold change)|≥ 0.585 and a FDR of 0.1) were not observed in 6-month-old mice (Fig. 2D). In contrast, in 17-month-old *Fa2h*^−/−^ mice, 21 proteins were significantly changed, among them only few established myelin proteins (Fig. 2E, Table 2). Notably, three myelin proteins showed a very similar, approximately 50% increase in 17-month-old *Fa2h*^−/−^ mice: Cadm4 (SynCAM4, Necl4), protein band 4.1G (Epb41l2), and Mpp6 (Pals2). The protein Lin7 (Lin7c) showed a similar increase (Fig. 2E), though this was statistically not significant. These four proteins are known to form a tetrameric complex in the membrane cytoskeleton of SLIs [31, 32]. The similar relative increase of all four proteins strongly suggests that they are mainly present in a complex in myelin, which is increased in the PNS of *Fa2h*^−/−^ mice.

**Table 2:** Proteins up- or downregulated in PNS myelin from 17-month-old *Fa2h*^−/−^ mice

Gene	Accession	Description	log(2)FC	*p*-value	adj. *p*-value
Mgll	O35678	Monoglyceride lipase	2.0417	0.00065	0.04408
Aoc3	O70423	Membrane primary amine oxidase	1.9773	0.00173	0.08416
Cd36	Q08857	Platelet glycoprotein 4	1.7469	0.00198	0.08434
Lyz1	P17897	Lysozyme C-1	1.5559	0.00001	0.00823
Lgals3	P16110	Galectin-3	1.2117	0.00004	0.01364
H2-K1	P01901	H-2 class I histocompatibility antigen. K-B alpha chain	1.0319	0.00106	0.06548
Slc27a1	Q60714	Long-chain fatty acid transport protein 1	0.9612	0.00285	0.09704
Epb41l2	**O70318**	**Band 4.1-like protein 2**	**0.7338**	**0.00014**	**0.03057**
Pals2	**Q9JLB0**	**Protein PALS2 / Mpp6**	**0.6964**	**0.00022**	**0.03057**
Cadm4	**Q8R464**	**Cell adhesion molecule 4**	**0.6916**	**0.00305**	**0.09890**
Anxa1	P10107	Annexin A1	0.6677	0.00052	0.04379
Fkbp3	Q62446	Peptidyl-prolyl cis–trans isomerase FKBP3	-0.6819	0.00261	0.09704
Ran	P62827	GTP-binding nuclear protein Ran	-0.7083	0.00050	0.04379
Cdv3	Q4VAA2	Protein CDV3	-0.7100	0.00145	0.07614
Plcb3	P51432	1-phosphatidylinositol 4.5-bisphosphate phosphodiesterase beta-3	-0.7561	0.00192	0.08433
Chmp1b1	Q99LU0	Charged multivesicular body protein 1b-1	-0.7998	0.00141	0.07614
Atp6v1c1	Q9Z1G3	V-type proton ATPase subunit C 1	-0.8616	0.00058	0.04379
Cast	P51125	Calpastatin	-0.9294	0.00271	0.09704
Sbds	P70122	Ribosome maturation protein SBDS	-1.0121	0.00022	0.03057
Tppp	Q7TQD2	Tubulin polymerization-promoting protein	-1.0368	0.00054	0.04379
Igkc	P01837	Immunoglobulin kappa constant	-1.4922	0.00284	0.09704

*Log(2)FC* log(2)-fold change (*Fa2h*^−/−^:*Fa2h*^+/+^), *adj p value* adjusted p value according to Benjamini and Hochberg [28]. Known myelin proteins (according to Table 1 in Siems et al. [28]) are highlighted in bold font

**Fig. 3:**
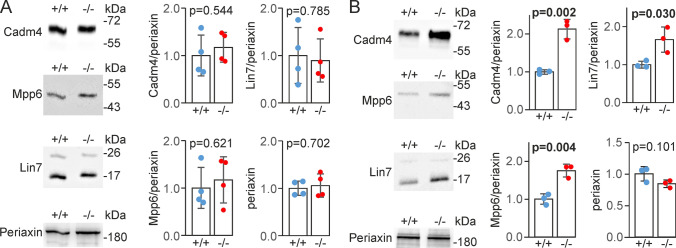
Cadm4, Mpp6 and Lin7 protein levels are in increased in old *Fa2h*^−/−^ mice. Western blot analysis of purified myelin from 4 to 6-month-old **A** and 17-month-old **B** *Fa2h*^+/+^ and *Fa2h*^−/−^ sciatic nerves. Blots were stained with the indicated antibodies and Western blot data from three or four independent samples (*n* = 3–4 mice per genotype) were evaluated by densitometry. Densitometric data were normalized to periaxin, except periaxin, which was not normalized. Data shown are the mean ± SD (*n* = 3–4) with the mean of wild type set to 1. *p* values in bold are significant (after control of the FDR at level 0.05)

Western blot analysis was used to confirm the mass spectrometry results using independent myelin samples isolated from sciatic nerves of young and old mice (Fig. 3). These experiments confirmed (1) unaltered levels of Cadm4, Lin7, and Mpp6 in young (4 to 6-month-old) *Fa2h*^−/−^ mice (Fig. 3a) and (2) a significant increase of Cadm4, Lin7, and Mpp6 in 17- to 18-month-old *Fa2h*^−/−^ mice (Fig. 3b) (we were unable to detect protein band 4.1G by Western blotting using commercially available antibodies).

### Increased Numbers of SLIs in Old but Not Young Fa2h^−/−^ Mice

**Fig. 4:**
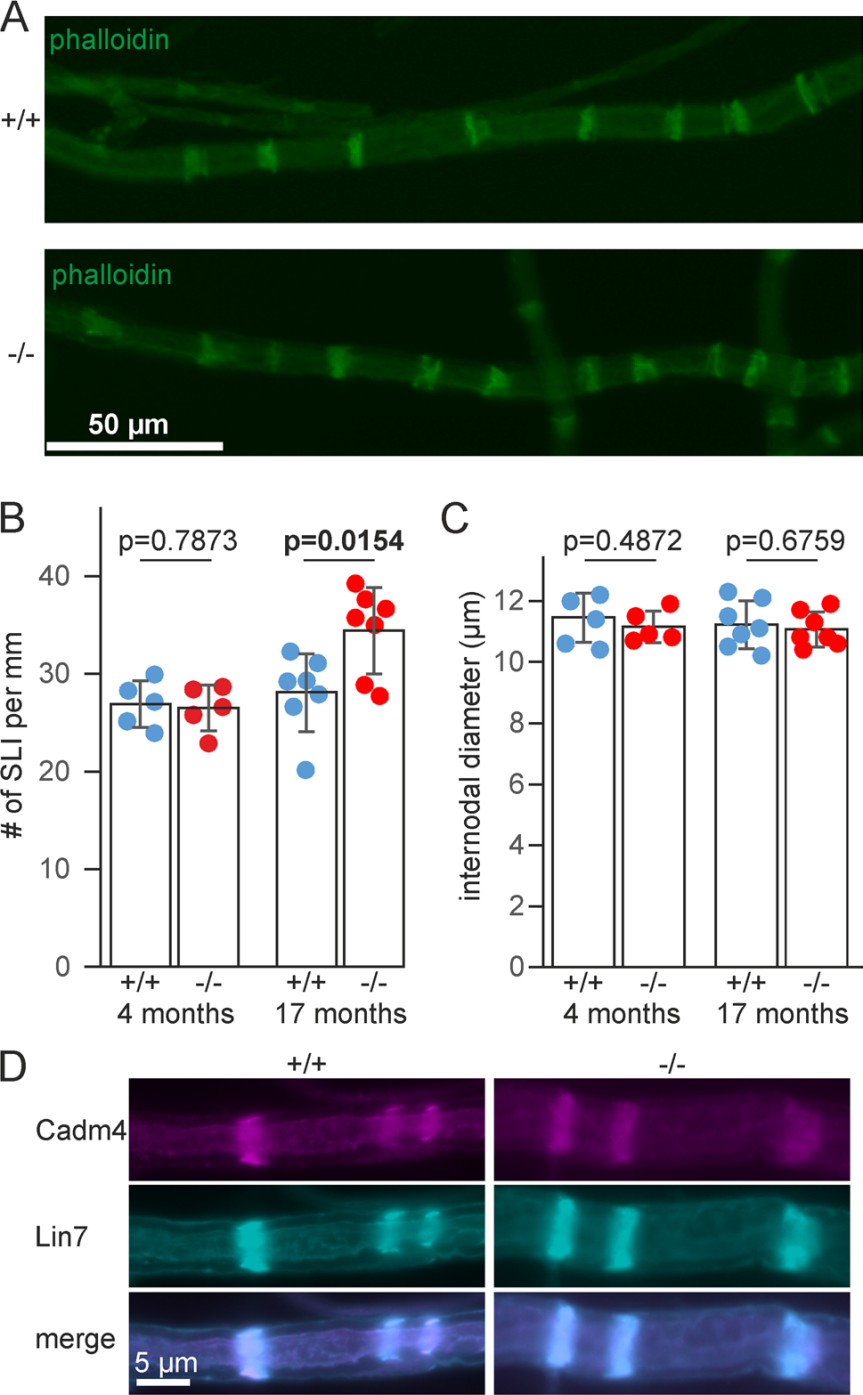
SLI frequency is increased in old *Fa2h*^−/−^ mice. **A** Phalloidin staining of teased fibers from 17-month-old *Fa2h*^+/+^ and *Fa2h*^−/−^ mice. **B** Quantification of SLI frequencies in teased fibers of sciatic nerves from 4-month-old (*n* = 5 mice per genotype; *N* = 50 fibers analyzed) and 17-month-old mice (*n* = 7 mice per genotype; *N* = 70 fibers). The number of SLIs was significantly increased in old but not young *Fa2h*^−/−^ mice. *p* value in bold indicates significant difference (t-test). **C** The average internodal diameter of axons examined to determine the SLI frequencies was not significantly different between genotypes. All data are shown as mean ± SD (*n* = 5–7) of the average SLI frequency or internodal diameter per mouse. **D** Immunofluorescence staining of Cadm4 and Lin7 in teased fibers from 17-month-old *Fa2h*^+/+^ and *Fa2h*^−/−^ mice. Both proteins co-localized and showed a similar distribution (mainly present in SLIs) in both genotypes

Potentially, the results from the proteome analysis could indicate an increase in the number of SLIs. On the other hand, however, other proteins known to be present in SLIs and identified by in our mass spectrometry screen were not increased in *Fa2h*^−/−^ mice (see Fig. 2E), and Western blot analysis showed no significantly altered levels of L-MAG (see Fig. 1c, d), which is also abundant in SLIs [33]. SLIs were quantified in teased fibers of sciatic nerves using fluorescently labeled phalloidin (Fig. 4a). The number of SLIs was significantly increased by 22% in 17-month-old (*p* = 0.0154, t-test) but not in young (4-month-old; p = 0.7873) *Fa2h*^−/−^ mice (Fig. 4B). Only axons with a comparable internode width were evaluated (Fig. 4C). The small but significant increase of SLI frequency was lower than expected from the relative increase of Cadm4, Lin7, Mpp6, and band 4.1G protein observed by mass spectrometry or Western blot analysis (≥ 50% increase). In line with their presence in the same molecular complex, Cadm4 and Lin7 colocalized in teased fibers and were mostly present in SLIs (Fig. 4D). We found no evidence for an altered distribution in *Fa2h*^−/−^ compared to *Fa2h*^+/+^ mice.

### Normal Length of Nodes of Ranvier and Paranodes in Fa2h^−/−^ Mice

**Fig. 5:**
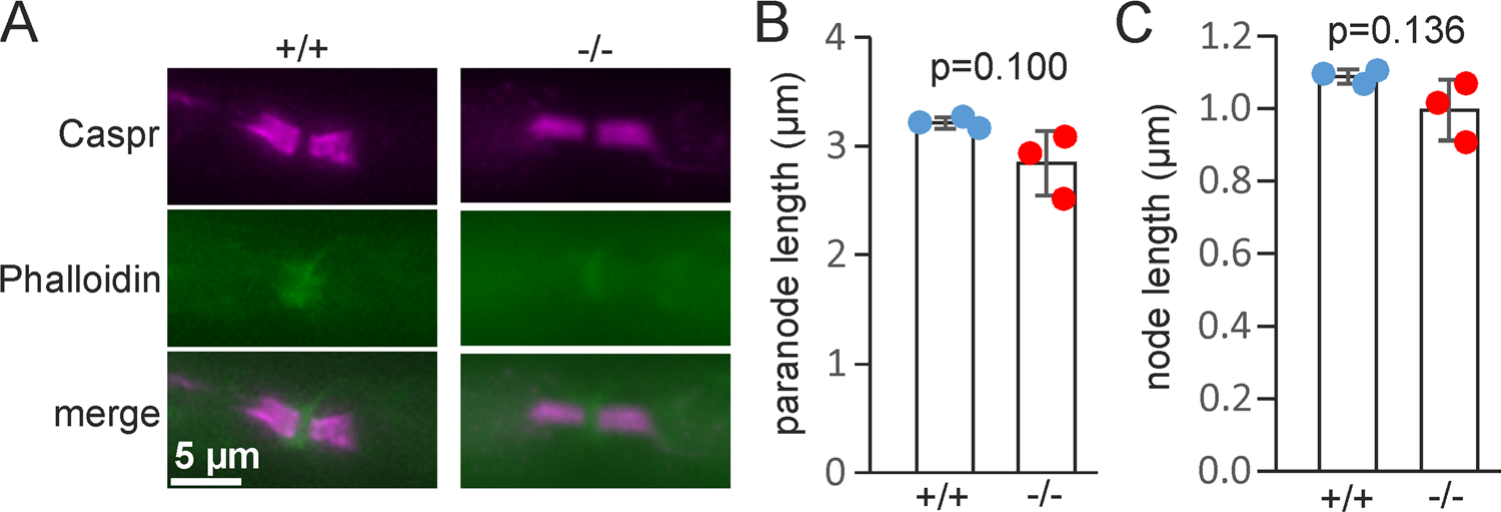
Length of paranodes and nodes of Ranvier are unaltered in *Fa2h*^−/−^ mice. **A** Teased fibers of sciatic nerves from 17-month-old *Fa2h*^+/+^ and *Fa2h*^−/−^ mice were stained with antibodies directed against Caspr (to label paranodes) and atto488-conjugated phalloidin to stain actin. The length of the paranodes (Caspr staining) **B** and the length of the nodes (distance between Caspr clusters) **C** were determined. No significant differences were observed. Data are shown with the mean ± SD of the average values obtained for each mouse analyzed (*n* = 3 mice per genotype; *N* = 30 nodes analyzed)

Because the paranodal structure is disturbed in mice lacking sulfatide [34], we wondered whether absence of 2-hydroxylated sulfatide may also affect the paranodes in older mice. Normal paranodal structure in young *Fa2h*^−/−^ mice was already demonstrated in a previous report [15]. We determined length of nodes of Ranvier in 17-month-old mice and paranodes using Caspr as paranodal marker [35] (Fig. 5a). No significant differences between genotypes were observed (Fig. 5b, c).

## Discussion

To our knowledge, the currently most comprehensive data set of mouse PNS myelin proteome used a gel- and label-free approach and identify 1083 proteins and could differentially analyze up to 700 proteins in the myelin proteome [29]. Using TMT-labeling for relative quantification, we were able to reproducibly identify and quantify 681 proteins (937 if proteins not detectable in all age groups were included). Thus, the number of identified and quantified proteins is comparable to previous proteome studies. The unique presence of several proteins in the proteome in our and the previous studies [29, 30] may be due to the different methods used, but the different ages analyzed (6 months and older in our study; 3–4 weeks in Patzig et al. [30] and Siems et al. [29]) may also contribute to these differences. Inconsistencies in the data of the 13-month samples that have prompted us to exclude them from further analyses and could be the result of improper TMT-labeling, though other errors in sample preparation cannot be excluded.

The differences between PNS myelin proteomes of *Fa2h*^+/+^ and *Fa2h*^−/−^ mice were small in all age groups examined, and upon focusing on well-established myelin proteins, we observed a rather specific increase of the four proteins Cadm4, Mpp6 (Pals2), Lin7, and band 4.1G (Epb41l2). These four molecules are co-regulated at the protein and mRNA level [29], and there is clear evidence that they form a complex in SLIs [32, 36–38]. Localization of this complex in SLIs depends on band 4.1G protein [39]. The almost identical relative upregulation of all four proteins by 50%, as observed in the mass spectrometric analysis strongly suggests that the four proteins are mainly present in this complex in myelin. Because several other myelin proteins known to be present in SLIs [32] were not found to be significantly increased in *Fa2h*^−/−^ sciatic nerve myelin, we assume that the increased level of the Cadm4 complex does not only reflect the higher SLI frequency. Whether increase in the membrane skeletal complex and the increase in SLI frequency are connected or independent events are unclear at present. A higher SLI frequency in *Mpp6*-deficient mice [31] indicates at least that changes in the level or localization of components of this membrane skeleton complex can affect SLI frequency, though the mechanism is currently not understood.

An increase in the number of SLIs together with structural abnormalities of the paranodes has also been observed in *Ugt8*- and *Gal3st1*-deficient mice, both lacking sulfatide [40–43]. In contrast to these mice, however, *Fa2h*^−/−^ mice, which lack only the 2-hydroxylated species of these lipids and have only slightly reduced total sulfatide levels in PNS myelin [15], have apparently normal paranodes at young [15] and old ages (this report). This indicates that sulfatide can fulfill its role at the paranodes irrespective of its hydroxylation status. In addition, the increased SLI frequency in sulfatide-deficient mice is age-independent [43]. We therefore assume that different mechanisms are responsible for the increase of the SLI frequency in *Fa2h*^−/−^ mice and such lacking sulfatide. The age-dependent increase in the number of SLIs correlates with the late onset of disease in *Fa2h*^−/−^ mice [15, 16]. Although PNS pathology is not a hallmark of hereditary spastic paraplegia, peripheral neuropathy has been observed in around 30% of SPG35 patients [14]. Because in remyelinated axons, the SLI frequency is increased [44, 45], it is possible that increased number of SLIs in *Fa2h*^−/−^ mice merely reflects remyelination. Peripheral nerves of 12-month-old *Fa2h*^−/−^ mice, however, showed thin myelin in less than 1% of axons, suggesting only a low level of de- and remyelination [16]. Therefore, the increased SLI frequency at 17 months may indicate significant peripheral neuropathy and demyelination in older *Fa2h*^−/−^ mice.

In a previous study with *Fa2h*^−/−^ CNS myelin, we could identify the oligodendrocytic myelin paranodal and inner loop protein (Opalin, Tmem10) to be significantly increased in myelin from old *Fa2h*^−/−^ mice [17]. Opalin is exclusively found in CNS myelin but not in PNS myelin [46, 47]. Furthermore, we found evidence for alterations in the transport and turnover of the protein, whereas expression of the Opalin gene was unaffected. These findings suggested that 2-hydroxylated sphingolipids may be required for correct sorting of Opalin and maybe other myelin proteins. Interestingly, Cadm4 was also increased in the CNS myelin proteome from 17-month-old mice according to our mass spectrometry analysis, although the null hypothesis could not be rejected in subsequent Western blot analyses [17]. Cadm4 also interacts with the choline transporter CTL1, and Cadm4-deficiency in Schwann cells leads to elevated levels of long chain and polyunsaturated phosphatidylcholine and phosphatidylinositol [48]. Therefore, elevated Cadm4 levels could potentially affect membrane lipid composition and membrane fluidity.

Because of its membrane topology, only Cadm4 could potentially be directly influenced by changes in the properties of galactosylceramide and sulfatide caused by absent 2-hydroxylation, as both lipids are only found in the extracellular leaflet of the plasma membrane. Further studies should examine if 2-hydroxylated sphingolipids may directly affect turnover and/or sorting of Cadm4 in myelinating Schwann cells. Although the increase of the Cadm4 complex in older mice correlates with the late onset of pathology in *Fa2h*^−/−^ mice [15, 16], it has to be examined whether increased levels of the Cadm4 containing complex in old *Fa2h*^−/−^ mice contributes to the pathogenesis of the disease and whether similar changes occur in human patients and may thus play a role in human SPG35.

## Supplementary Information

Below is the link to the electronic supplementary material.

Supplementary file1 (PDF 707 KB)

Supplementary file2 (XLSX 344 KB)

Supplementary file3 (DOCX 12 KB)

## Data Availability

The mass spectrometry proteomics data have been deposited to the ProteomeXchange Consortium via the PRIDE [25] partner repository with the dataset identifier PXD030244 and 10.6019/PXD030244.

## Abbreviations

Cadm4: Cell adhesion molecule 4
CNP: 2',3'-Cyclic nucleotide 3'-phosphodiesterase
Epb41l2: Erythrocyte membrane protein band 4.1 like 2
FA2H: Fatty acid 2-hydroxylase
FDR: False discovery rate
LC–MS/MS: Liquid chromatography-tandem mass spectrometry
Lin7: Lin-7 homolog
L-MAG: Large isoform of myelin-associated glycoprotein
MBP: Myelin basic protein
Mpp6: Membrane protein, palmitoylated 6/MAGUK p55 subfamily member 6
Opalin: Oligodendrocytic myelin paranodal and inner loop protein
Pals2: Protein associated with LIN7 2
SD: Standard deviation
SLI: Schmidt-Lanterman incisure
SPG35: Spastic paraplegia type 35
TMTsixplex: Tandem mass tag 6-plex
