# Supplementary material for: Age-Dependent Increase in Schmidt-Lanterman Incisures and a Cadm4-Associated Membrane Skeletal Complex in Fatty Acid 2-hydroxylase Deficient Mice: a Mouse Model of Spastic Paraplegia SPG35
Source: Mol Neurobiol. 2022 Apr 20;59(7):3969–79. doi: 10.1007/s12035-022-02832-4 (PMC9167166; doi:10.1007/s12035-022-02832-4)
Supplement: Supplementary file 1 — Supplementary file1 (PDF 707 KB) [file 12035_2022_2832_MOESM1_ESM.pdf]

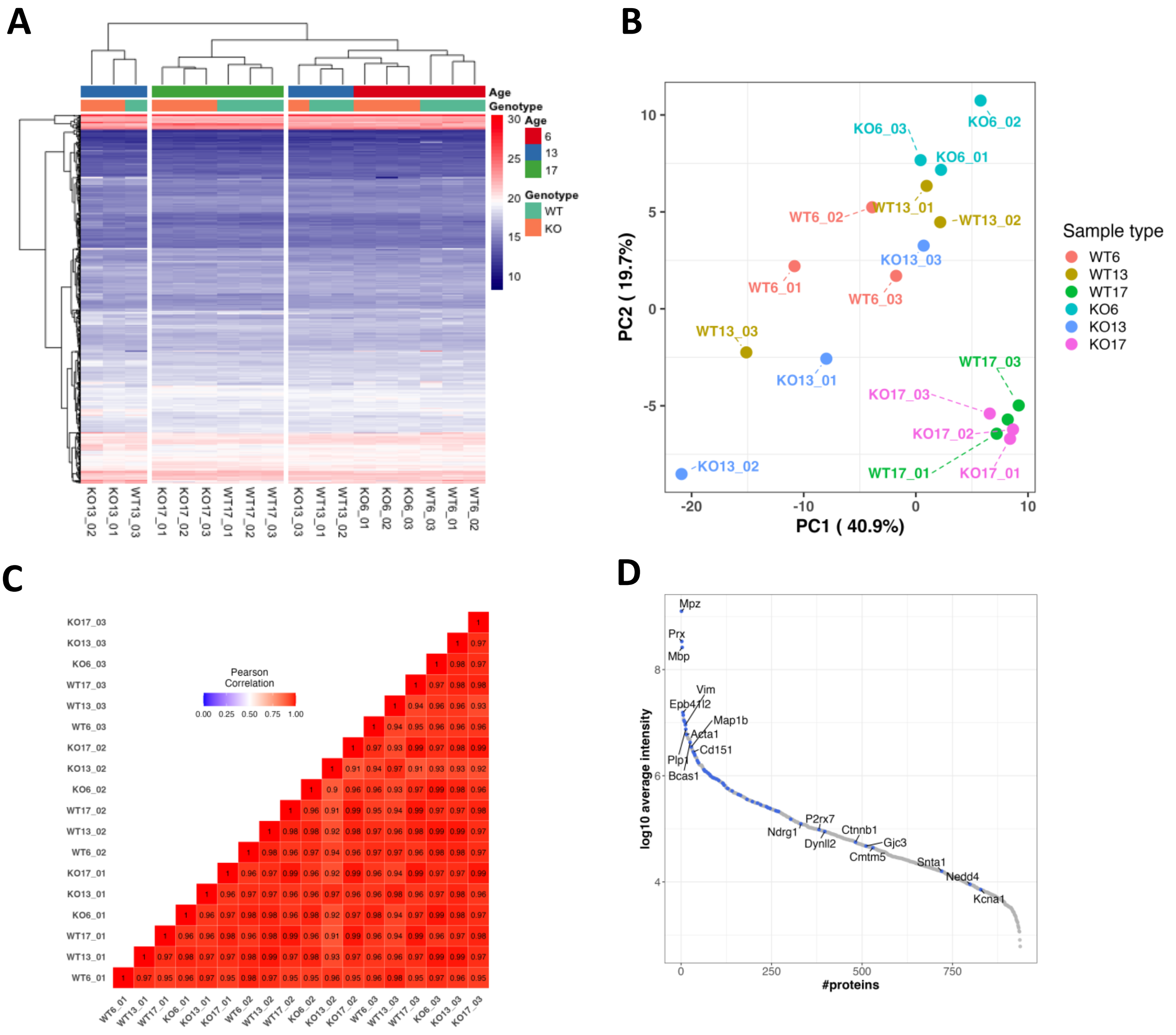

Figure S1: A. Clustered heatmap of normalized log2 protein abundances in PNS myelin (distance = Manhattan, clustering = complete). Genotype and animal age (month) is indicated by color markers. Missing values are plotted in dark grey. **B.** Principal component analysis of normalized log2 protein abundances. Different sample types are indicated by color coding. **C.** Pearson correlation plot of normalized log2 protein abundances. Correlation coefficients are given as printed numbers and indicated by color coding. **D.** Protein abundance plot showing the log10 intensity for all 937 proteins that have been identified in at least two replicates, averaged over all samples. Known myelin proteins are highlighted in blue and a selection is labeled with their respective gene name. KO = *Fa2h*$^{-/-}$, 6 = 6 months, 13 = 13 months, 17 = 17 months.
