# Supplementary material for: Age-Dependent Increase in Schmidt-Lanterman Incisures and a Cadm4-Associated Membrane Skeletal Complex in Fatty Acid 2-hydroxylase Deficient Mice: a Mouse Model of Spastic Paraplegia SPG35
Source: Mol Neurobiol. 2022 Apr 20;59(7):3969–79. doi: 10.1007/s12035-022-02832-4 (PMC9167166; doi:10.1007/s12035-022-02832-4)
Supplement: Supplementary file 3 — Supplementary file3 (DOCX 12 KB) [file 12035_2022_2832_MOESM3_ESM.docx]

**Link to supporting data**

Reviewer account details for access to the proteome data set via the PRIDE partner repository with the dataset identifier PXD030244 and 10.6019/PXD030244 are as follows:

Website: <https://www.ebi.ac.uk/pride/login>

Username: reviewer_pxd030244@ebi.ac.uk

Password: 2azRDJT2
